# Mitigating Stress and Supporting Health in Deprived Urban Communities: The Importance of Green Space and the Social Environment

**DOI:** 10.3390/ijerph13040440

**Published:** 2016-04-22

**Authors:** Catharine Ward Thompson, Peter Aspinall, Jenny Roe, Lynette Robertson, David Miller

**Affiliations:** 1 OPENspace Research Centre, University of Edinburgh, Lauriston Place, Edinburgh EH3 9DF, UK; 2 School of Energy, Geoscience, Infrastructure and Society (EGIS), Heriot Watt University, Edinburgh EH14 4AS, UK; p.a.aspinall@hw.ac.uk; 3 Center for Design and Health, University of Virginia, Charlottesville, VA 22904, USA; jjr4b@virginia.edu; 4 Stockholm Environment Institute, University of York, Heslington, York YO10 5DD, UK; 5 Mackintosh School of Architecture, Glasgow School of Art, Renfrew St., Glasgow G3 6RQ, UK; L.Robertson@gsa.ac.uk; 6 The James Hutton Institute, Aberdeen AB15 8QH, UK; David.Miller@hutton.ac.uk

**Keywords:** urban green space, stress, health, socio-economic deprivation, social isolation, place belonging, physical activity, gardens, allotments

## Abstract

Environment-health research has shown significant relationships between the quantity of green space in deprived urban neighbourhoods and people’s stress levels. The focus of this paper is the nature of access to green space (*i.e.*, its quantity or use) necessary before any health benefit is found. It draws on a cross-sectional survey of 406 adults in four communities of high urban deprivation in Scotland, United Kingdom. Self-reported measures of stress and general health were primary outcomes; physical activity and social wellbeing were also measured. A comprehensive, objective measure of green space quantity around each participant’s home was also used, alongside self-report measures of use of local green space. Correlated Component Regression identified the optimal predictors for primary outcome variables in the different communities surveyed. Social isolation and place belonging were the strongest predictors of stress in three out of four communities sampled, and of poor general health in the fourth, least healthy, community. The amount of green space in the neighbourhood, and in particular access to a garden or allotment, were significant predictors of stress. Physical activity, frequency of visits to green space in winter months, and views from the home were predictors of general health. The findings have implications for public health and for planning of green infrastructure, gardens and public open space in urban environments.

## 1. Introduction

A growing body of evidence shows a relationship between levels of green space in the local neighbourhood and people’s health and wellbeing, especially for low-income and deprived urban or suburban populations [1,2,3]. Studies in Japan have shown that access to forest environments can promote lower concentrations of cortisol, lower pulse rate and blood pressure, greater parasympathetic nerve activity and lower sympathetic nerve activity compared to city environments [4,5,6]. These studies suggest that green space may offer opportunities to buffer or mitigate health outcomes for urban populations. Recent research in the United Kingdom (UK), using objective, GIS-based measures of the amount of green space in the residential environment and biomarkers of stress combined with self-report measures, has shown a significant association between the amount of green space in the environment and stress levels for a deprived urban population not in work [7,8].

Such findings offer evidence that underlines the potential importance of access to green space when developing future strategies for healthier urban environments such as the Scottish Government’s Place Standard [9]. Studies have attempted to assess how much green space is needed for healthy functioning of a city’s ecological systems [10] and to consider the role of ecosystems services for human health [11]. However, questions remain regarding the quantity and types of green space within a community which are associated with significant differences in stress levels and with human health more generally [12].

In a study of green space quality and quantity in relation to self-reported general health and certain mental health outcomes, in four large Dutch cities, Van Dillen *et al.* [13] showed that quality measures may add predictive value to quantity measures. Nonetheless, quantity and quality were related to a substantial degree. However, the study also identified the importance of street trees and their contribution to views of greenery, found to be associated with health outcomes. This suggested that measures of green space relevant to health in urban areas should include not just dedicated recreational space such as public parks and playing fields but also other types of green space, such as road verges. A study of allotment gardening by van den Berg and Custers [14], using salivary cortisol as a measure of stress, found that a period of gardening led to greater levels of stress reduction than a restful indoor task (e.g., reading), adding support to evidence that access to a garden or allotment offers health benefits in general, and benefits for buffering stress [15] in particular (An allotment is a plot of land rented by an individual for growing vegetables or flowers. Provision of groups of allotments, often owned by urban authorities, is commonplace in many parts of Europe and can be an important green space and gardening resource for urban dwellers.).

A further consideration is the area around a person’s home in which the contribution of green space may be significant with respect to their health. Maas *et al.* [2] used a radius of 1 km and 3 km, respectively, around people’s homes to study the effect of the quantity of green space within the living environment on a range of physical and mental health measures. They found that green space close to home is more important, although a similar study on green space as a buffer between stressful life events and health only found an effect within the wider area of 3 km [16]. However, more local green space measures have also been shown to be relevant. The study by van Dillen *et al.* [13] measured the quantity of green space within an area of radius 500 m around participants’ homes and identified links between green space quantity and self-reported health. The European Common Indicator of availability of local public open areas does not set a target and is not specifically focused on green space, but is based on the percentage of citizens living within 300 m from a public open area of minimum size 0.5 hectares [17]. This is considered the approximate distance within a 5-min walk from home, taking into account varying walking speeds of different age groups and barriers such as main roads or indirect routes that may add time to any distance walked.

While useful as a basis for research, such measures of proximity of green space do not necessarily equate with visibility, perceptions or use of that space for local residents. Research in Scotland has shown that, for many people, regular green space use is not limited to locations within 300 m from home [18] and perceptions of green space proximity and accessibility may relate to what is perceived to be part of the neighbourhood or available for community use, rather than objectively measured distance [19]. On the issue of green space visibility, the scientific literature on aspects of stress relief and attention restoration [20] offered by green space suggests that simply viewing green space may offer a range of health benefits [21,22]. It is important therefore to consider whether merely viewing green space from one’s home or street is sufficient to be associated with lower stress levels, or whether visiting green space is necessary to gain significant benefit. Equally, it is important to understand whether access to a private garden or allotment offers sufficient green space experience to be associated with lower perceived stress levels, as van den Berg and Custers [14] suggest, compared with access to a park or other publicly accessible open space.

A relevant consideration in studying links between green space and health is the former’s potential role as an environment conducive to physical activity or social wellbeing. The importance of physical activity for health, and the role of physical environment in supporting activity, has been understood for some time and underlined in recent research reviews [23]. Recognition of the importance of social capital for health, both physical and mental, is a more recent phenomenon [24], with social isolation a particular health risk for older people [25,26,27]. Heinrichs *et al.* [28] have shown how social contact can have positive effects on mood and stress level. Choenarom *et al.* [29] have demonstrated the role of a sense of belonging and social support for perceived stress and its links to depression and [30] demonstrated an inverse relationship between a sense of belonging and perceived stress.

A considerable body of research has reported on environmental support for physical activity, and walking in particular [31,32]. However, evidence shows that socio-economic deprivation also plays an important role in such relationships between green space and health. Lachowycz and Jones’s study of a large UK sample [32] confirmed an association between green space access and reduced cardiovascular mortality found previously [1,33] but only amongst the most socio-economically deprived groups. Although a relationship between green space access and walking was found for all areas, the authors found no evidence that recreational walking explained the relationship between green space access and mortality. The authors suggest that the relationship may be explained by mediators other than walking, such as psychosocial factors. Maas *et al.* [2] studied the relationship between lower morbidity and a green living environment in The Netherlands and found the relationship was strongest for anxiety disorder and depression and stronger for children and people of lower socio-economic status. Another UK study [34] identified significant associations between reported access to, and better quality of, green space and reduced psychological distress in a deprived urban population. Such evidence is supported by a European epidemiological study [3] of associations between mental wellbeing and people’s level of financial strain; it found that socioeconomic inequality in mental wellbeing was 40% narrower among respondents reporting good access to green space, compared with those with poorer access.

One way in which good access to green space may contribute to reduced health inequalities in income-deprived communities is through frequency of and/or time spent in outdoor activities, which may in turn support social engagement and thereby link to wellbeing [18,35]. In a Dutch study, de Vries *et al.* [36] found an association between streetscape greenery and perceived social cohesion at the neighborhood scale. Maas *et al.* [35] considered loneliness, social support, and contact with neighbours and friends in the neighbourhood as potential mediators of links between green space quantity and self-reported health outcomes. They found, overall, that people with more green space in their living environment felt less lonely and less lacking in social support, but they did not report having more contact with neighbours or friends in the neighbourhood or receiving more social support in practice. The authors suggest that the relationship between green space and social contacts has more to do with the fact that green spaces can strengthen a sense of community via residents developing their attachment to place and place identity, than with any greater contact with neighbours.

In their review of green space links with health, Hartig *et al.* [37] emphasise that relationships between social wellbeing and greenspace are complex and not easily explored via experimental research. People’s social wellbeing may be detrimentally affected by green and open space in which they feel unsafe or where other people engage in unwelcome or anti-social behaviour. This may be particularly true for areas of socio-economic deprivation. There is also considerable evidence that women experience fears for personal safety differently from men and that this influences how green space is perceived and used [38,39,40], which may in turn reflect differences by sex in the associations between green space and health [41]. Jiang *et al.* [42] have shown differences between men and women in relationships between urban tree cover and stress levels and Roe *et al.* [8] found differences by sex in patterns of stress associated with local green space in deprived urban areas in Scotland.

Drawing on the above research, our study attempts to investigate what level or type of availability of green space is associated most strongly with any difference in perceived stress levels, and in general health. It also explores potential pathways for explaining links between green space and stress levels. As the association between green space and stress or mental wellbeing has been shown to be strongest in low socio-economic groups [2] or populations suffering financial strain [3], as described above, our study has focused on economically deprived urban populations.

The aims of the study are to investigate:

(a) What measures of quantity or use of local green space are associated with differences in stress levels in deprived urban communities?
(b) Are physical activity or social wellbeing variables also associated with differences in stress levels in deprived urban communities, and how do these relate to green space variables?
(c) Are any relationships between quantity or use of local green space and stress also found for general health as an outcome?
(d) Do physical activity or social wellbeing variables play a role in predicting general health?

## 2. Materials and Methods

### 2.1. Study Design

This study is based on a cross-sectional questionnaire survey of adults living in areas of socio-economic deprivation, as defined by the Carstairs Index [43], with varying levels of green space, located in two Scottish cities. It is part of a wider project, GreenHealth [44], in which the criteria used for initial identification of study sites across the project were based on the most recent (2001) national decennial population census data and electoral wards available at the time of data collection. To identify areas of socio-economic deprivation, Carstairs Index figures were used in preference to the Scottish Index of Multiple Deprivation (SIMD) as the latter combines seven domains that include measures of health and social and physical environment which we wanted to investigate separately. Carstairs scores are an index of deprivation at ward level based on an unweighted combination of four census variables: unemployment, overcrowding, car ownership and low social class. A higher score equates with higher deprivation, with a score of greater than 6 indicating “very deprived areas”. In the 2001 population census for Scotland, the mean was 0 (SD = 3.6). Four areas (two in each city) were chosen based on the Carstairs Index and an objective measure of green space, derived from satellite imagery and summarised by census wards in each city [41,45], using 2001 data available at the Centre for Research on Environment Society and Health (CRESH) [41,46]. These data include parks, woodlands, scrub and other natural environments, but do not include private gardens. 

### 2.2. Choice of Study Sites

**Figure 1:**
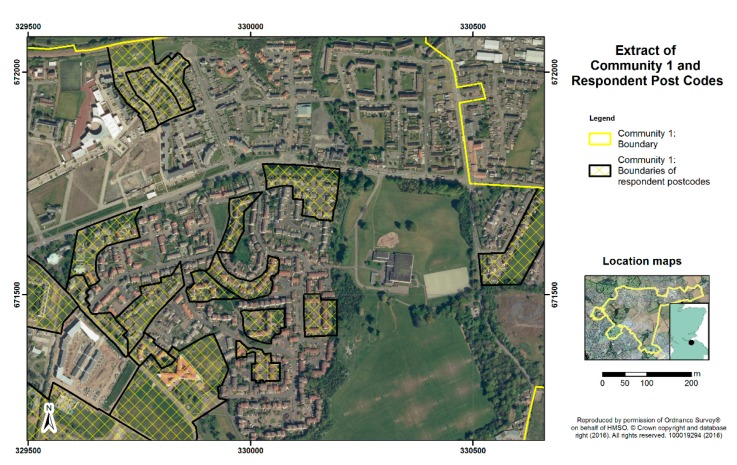
Aerial view of typical residential neighbourhood in Community 1.

**Figure 2:**
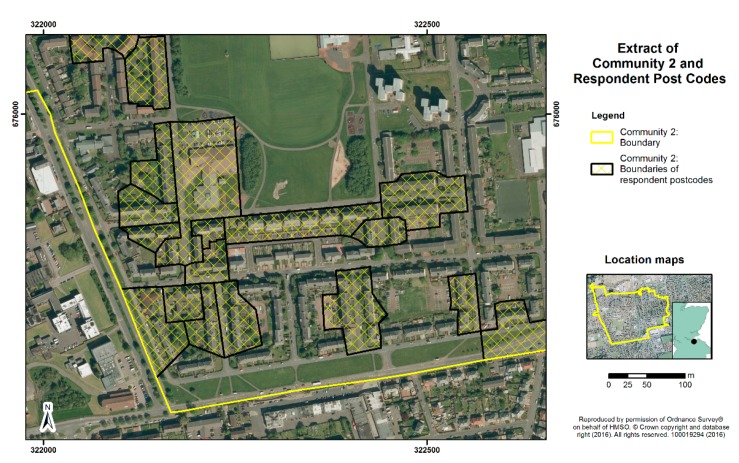
Aerial view of typical residential neighbourhood in Community 2.

**Figure 3:**
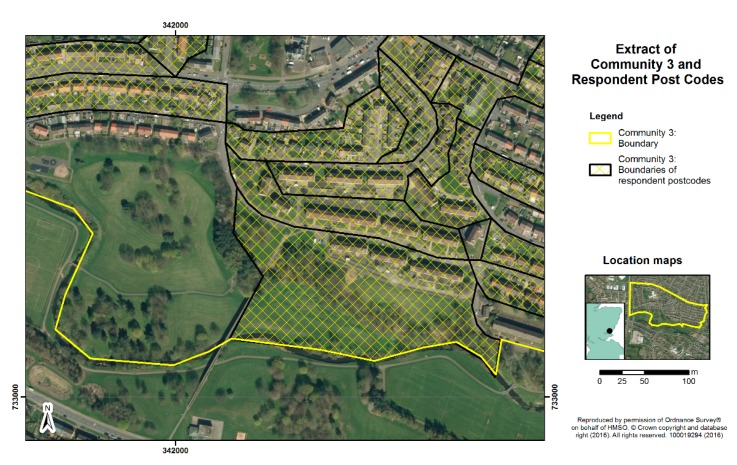
Aerial view of typical residential neighbourhood in Community 3.

**Figure 4:**
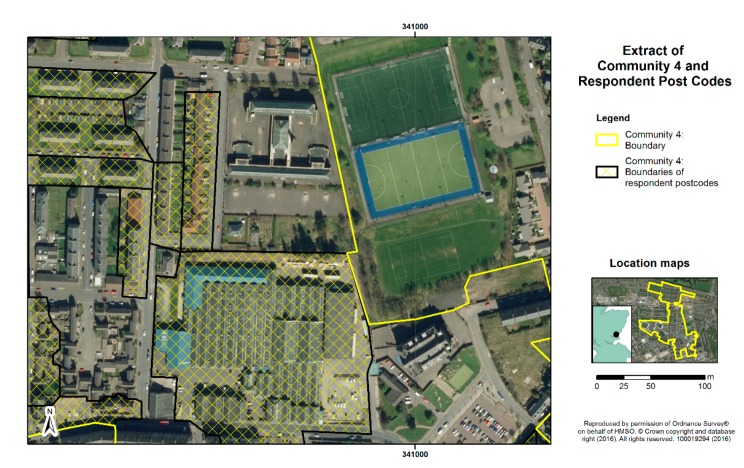
Aerial view of typical residential neighbourhood in Community 4.

Consideration of sites for the survey was based on the above criteria, matching high levels of deprivation with as wide as possible a variation in green space quantity in the residential area, based on census ward measures. The sites chosen within Edinburgh (Communities 1 and 2) and Dundee (Communities 3 and 4) comprised postcode areas with mean Carstairs scores over 3.6, indicating they are among the 17% most deprived areas of Scotland. One site in each city (Communities 1 and 3) had public green space levels of over 65%, and one site in each (Communities 2 and 4) had green space levels below 35%, based on ward level data and boundaries. Figure 1, Figure 2, Figure 3 and Figure 4 display the typical neighbourhood in each community and provide an impression of the patterns of green space compared to housing and other characteristics of built form.

Communities 1 and 2 are principally constituted by social rented housing, initially built in the 1930s to house populations from clearance of slum properties in the centre of Edinburgh, displaced to the urban periphery. Community 1 initially housed a substantial workforce for local breweries and mines; however, the population fell steeply with the decline of the economy during the 1960s and 1970s and resulting socio-economic problems led to its identification as the fourth most deprived area in Scotland. There have been successive regeneration initiatives in these communities since the 1980s but social and economic problems remain such that both communities contained SIMD zones among the 5% and 10% most deprived in Scotland, as of 2009 [47].

Communities 3 and 4, in Dundee, also contain significant areas of social rented housing. Community 3 was initially constructed in the late 1940s and 1950s, although some regeneration also took place in the 1980s. It is on the northern periphery of Dundee, and contains SIMD zones within the 15% and 20% most deprived in Scotland. Community 4 is in an area originally developed to house textile workers under the 19th century industrial revolution. It is comparatively close to the city centre and contains zones within the 5%, 10% and 15% most deprived in Scotland. It has undergone regeneration since the turn of the millennium.

### 2.3. Recruitment of Sample

Approximately 100 participants were recruited from each community using postcodes from within each community. Each study community had a total population of approx. 5000. A stratified sampling methodology was used that matched proportions of the sample to those of the census ward (based on the 2001 national census) for each case study area, based on age, gender and socio-economic group. The survey was administered face-to-face by a professional survey company in May and June 2010.

### 2.4. Measures

#### 2.4.1. Individual Level Health and Wellbeing Variables

Three health variables were potentially available for the study reported here: self-reported stress based on the Perceived Stress Scale (PSS) [48]; mental wellbeing, based on the Shorter Warwick-Edinburgh Mental Wellbeing Scale (SWEMWBS) [49]; and a single-item assessment of general health. However preliminary analysis showed, firstly, that there were two latent classes of health and, secondly, that the strongest association within the variables was between stress and wellbeing (*r* = −0.42, *p* < 0.001). As a consequence, the primary outcome measures selected for this study were self-reported stress and general health. Stress was measured using PSS, comprising 10 items (e.g., feeling nervous and stressed; feeling on top of things; being angered because of things outside your control) measured on a 5-item response from “never” to “very often”. The final score assesses perceived stress over the preceding month and can range from 0 (minimum level of stress) to 40 (maximum level of stress). To measure overall health, a single item asked participants to rate their general health, ranked on a 5-category Likert scale from 1 (very poor health) to 5 (very good health).

Secondary measures were physical activity levels and social wellbeing, all based on self-report. Physical activity levels were measured using one item asking for the number of days on which physical activity (of sufficient exertion to raise breathing rate) reached or exceeded 30 min, recalled over the preceding 4 weeks. This item is recommended by the British Heart Foundation National Centre [50].

Social wellbeing was based on three aspects: place belonging, (“how strongly do you feel you belong to your neighbourhood or local area?”) ranked on a 5-item scale from “strongly disagree” to “strongly agree”; social isolation (“how often do you feel that you lack companionship?”), ranked on a 3-item scale of “often”, “some of the time” or “hardly ever”; and neighbourhood trust (how comfortable giving your home key to a neighbour to keep an eye on while you are on holiday), ranked on a 4-item scale from “very uncomfortable” to “very comfortable”.

#### 2.4.2. Other Individual Characteristics

The other characteristics measured via the questionnaire included sex, age, educational level, relationship status (married, cohabiting with partner, single, *etc.*), private car access, whether there are children under 16 in the household, and employment status.

#### 2.4.3. Area-Level Deprivation

The Carstairs Index score for the postcode of each participant was also noted. Most participants were in locations with a Carstairs score of 7.1 (Community 1), 8.7 (Community 2), 3.7 (Community 3) or 5.9 (Community 4), but the location of some participants was in an adjacent census zone. Therefore, the Carstairs scores for each participant’s location were recorded for analysis.

#### 2.4.4. Self-Reported Access to Green Space

Survey participants were asked questions about their access to local green space. In order to keep participant burden low, we relied on self-reported “access” for these variables, and did not ask respondents to locate the green space in question on a map. Questions included: how often they visit their nearest green space in winter months (October to March) and summer months (April to September), on a five-point scale from “every day” to “never”; if they had a garden or allotment (yes/no); and if they have a view to green space or hills from their home (yes/no).

#### 2.4.5. Objective Measures of Green Space

**Figure 5:**
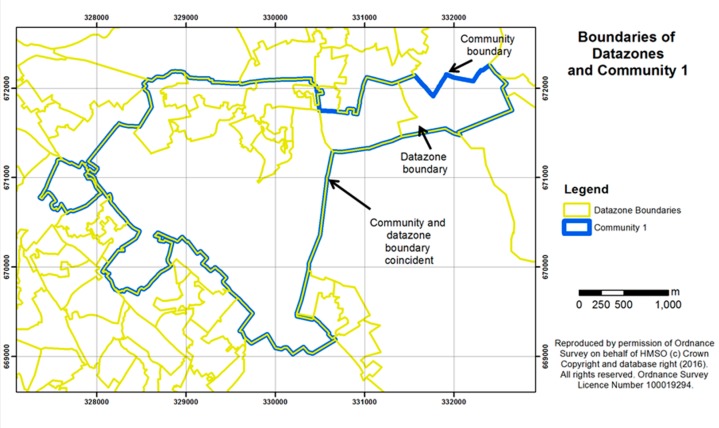
Community 1 outline showing boundaries of the datazones used for calculating the percentage area of green space per survey participant.

**Figure 6:**
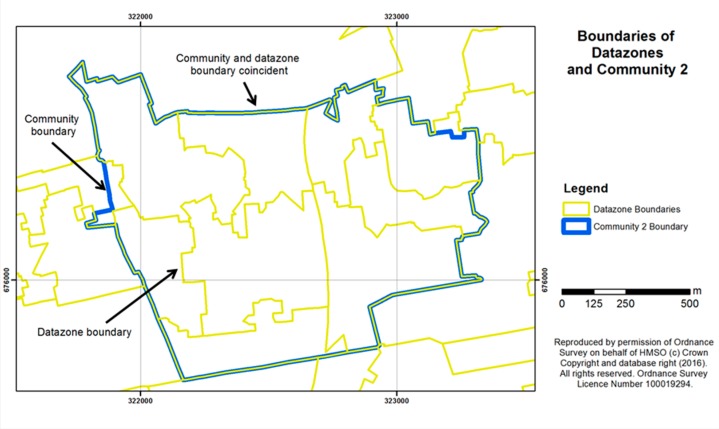
Community 2 outline showing boundaries of the datazones used for calculating the percentage area of green space per survey participant.

The study sites had initially been chosen as part of the wider GreenHealth project on the basis of the percentage area of public green space at census ward level. However, closer analysis of the data at a more detailed scale revealed considerable variation in quantities of green space around the living area of each participant, and different levels of access to gardens (some of which might be shared between residents of tenement blocks of flats/apartments but still private rather than public). It was recognised that the ward level data, *i.e.*, the area of green space within census ward boundaries, was not detailed enough to take into account more local neighbourhood variations in housing layouts and green space patterns. A separate, comprehensive measure of green space was therefore developed at a finer scale, reported as a percentage of the area of the datazone within which each participant lived. Datazones were defined in Scotland in 2004 as a standardised unit for statistical reporting and contain a number of postcode areas but are smaller than the 2001 census wards. They have populations of between 500 and 1000 household residents, taking into account physical boundaries and natural communities so that, as far as possible, they contain households with similar social characteristics. Figure 5 and Figure 6 show examples of the datazone boundaries for Communities 1 and 2, indicating the zones within which green space levels were mapped for each community. The figures also indicate that the community boundaries from which our sample postcodes were taken (based on the 2001 census wards) do not always align exactly with the later-derived datazone boundaries. Nonetheless, for each participant we used the measure of percentage green space area in the datazone in which they resided, as this provided the finest available spatial resolution of green space.

The datazone green space measure was based on reclassifications of the Ordnance Survey MasterMap and a city-wide audit of greenspace for Edinburgh, using classifications under Scottish Government’s 2008 Planning Advice Note on Planning and Open Space (PAN 65) [51] and cross-referencing to Scotland’s Greenspace Map [52]. Thus the green space data were based on more recent mapping and verification of land use (post-2008) than the census ward level data, and at a finer resolution. The *percentage green space area* derived by this means included public green space, private gardens, and other green space such as roadside trees and grass, but did not include woodland or forestry areas that were publicly inaccessible. The mean green space area for participants across all communities was 56.83% (SD = 12.34).

### 2.5. Characteristics of the Sample

Of the total sample of 406 participants, 184 (45.3%) were male and 222 (54.7%) were female. Age ranged from 16 to 87 years, with a mean age of 44 years (SD = 17.1 years). Carstairs deprivation scores ranged from a minimum (least deprived) of −1.3 to a maximum of 8.7, with a mean of 6.2 (SD = 2.3). Table 1 summarises the characteristics of the sample in total and by community.

### 2.6. Approach to Analysis

Given the statistically significant variations in perceived stress and general health by community (both Kruskal-Wallis *p* < 0.001) indicated in Table 1, it was deemed appropriate to consider whether there were sub groups in the sample in relation to our outcome measures. Conventional regression analysis implicitly assumes that the predictors from a sample can be generalized to the population. However in situations where the sample contains sub-populations this assumption is unrealistic and conventional regression may produce biased estimates [53]. In such circumstances the use of regression mixture (or latent class) models provides greater flexibility in allowing regression parameters to vary within each sub-class within the data.

The CHAID (Chi-squared Automatic Interaction Detection) algorithm provides one such approach by profiling latent segments in a tree structure which can include classification for nominal or ordinal dependent variables [54]. CHAID also allows for more than two branchings at any point in the tree at which there are significant differences. When compared with other inferential statistical methods, the CHAID algorithm has additional advantages. First, as Green and Salkind [55] point out, CHAID does not require the assumptions for running typical inferential statistical analysis. Second, while inferential statistics evaluate only whether there exists a significant difference among mean scores of dependent variables in each category of independent variables, the CHAID algorithm makes decisions about dependent variables at each terminal node as the tree progresses.

In the approach taken here, a CHAID analysis was initially applied separately to each of two outcome (dependent) variables—stress and general health—to identify possible heterogeneous segments in the data between communities. Once the main segments in the sample were identified, this was followed for each segment by regression analysis, using stress or general health as the dependent variable and independent variables based on the measures identified above, *i.e.*, physical activity and social wellbeing variables, individual characteristics, area-level deprivation, self-reported access to green space and the objective measure of green space in the neighbourhood.

We used a new form of high dimensional Correlated Component Regression (CCR) with M-Fold Cross Validation. CCR has a number of benefits in addition to its capacity to deal with multicollinearity. It is one of a number of methods which have been developed to regularise regression for linear modelling (although, so far as we know, it is the only regularisation model available for non-linear models such as logistic regression) as a means of reducing prediction error. Regularisation is achieved through component/dimension reduction strategies, as shown by Magidson [56] and applied, for example, in clinical research contexts [57,58]. The purpose is to optimise R squared by manipulating both the number of correlated components (k) and the predictors (p) in any model. Whereas in conventional regression, k is fixed at k = p, Magidson has shown that this saturated model fit is frequently improved when k < p, leading to more parsimonious models with less variance in estimates over different samples. In addition, the usually recommended limits on the number of cases per predictor are relaxed to the extent that (p), the number or predictors, can exceed (n), the number of cases—hence, its definition as high dimensional. Another unique feature of CCR is that *p* values are replaced by cross-validated, out-of-sample performance as provided in the predictor tables. Thus, through its regularisation process, it prevents model over-fit while delivering better out of-sample prediction in cross validation. The analysis also enables suppressor variables to be identified.

In determining the final number of predictors, a standard error rule was applied. This means that either the optimum number of predictors was selected or, in situations where smaller numbers of predictors fell within one standard error of the optimum, then the predictor number closest to one standard error from the optimum was selected *i.e.*, a parsimonious solution. To assess the relative importance of each of the predictor variables to the dependent variable, the Pratt measure was used as this compensates for collinearity between correlated predictors in beta weight estimations [59]. In Pratt’s importance measure, the unique contribution of any predictor to the final regression model is obtained by multiplying its beta weight by its zero-order correlation with the dependent variable (*i.e.*, its correlation with the dependent variable in isolation from other predictors).

## 3. Results

### 3.1. Predicting Stress

#### 3.1.1. Segments in the Sample

**Figure 7:**
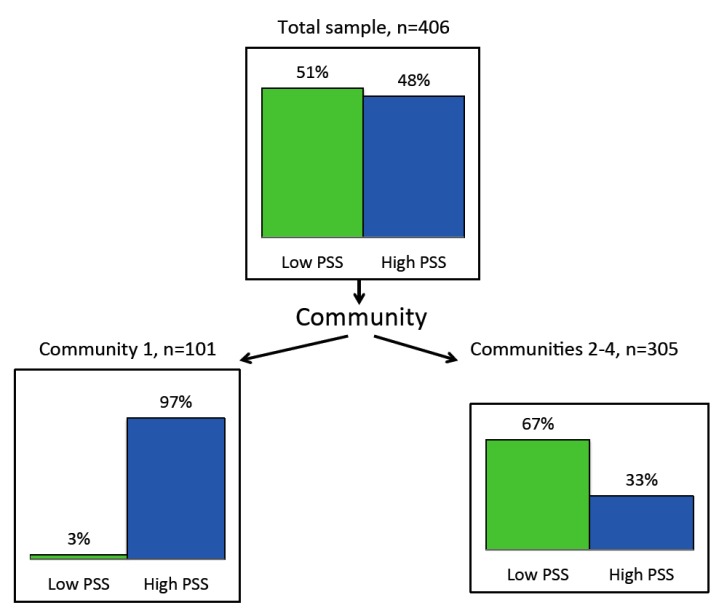
CHAID segmentation of sample by stress (PSS).

The results of CHAID applied to PSS as a dichotomised variable for the sample as a whole (*n* = 406) are shown in Figure 7. The top box shows an approximately even distribution of stress across the sample, with 51.2% in the lower stress group and 48.8% in the higher stress group (dichotomised around a median score of 15). In Community 1 (*n* = 101), 97.0% are in the higher stress group, while in Communities 2, 3 and 4 (*n* = 305), only 32.8% are in the higher stress group. The CHAID split is very highly significant (χ^2^ = 149.7, df = 1, *p* < 0.0001).

#### 3.1.2. Predictors of Perceived Stress in Community 1

The CCR logistic regression model for Community 1 (*n* = 101) used PSS as the dependent variable and independent variables as identified earlier. In determining the final number of predictors, as explained in Section 2.6, a standard error rule was applied to produce a parsimonious solution.

The CCR regression resulted in a single predictor model based on whether or not participants had *a garden or allotment.* The key variables predicting stress arising from this and subsequent regression models used in the analyses, undertaken according to the procedures described in Section 2.6, are summarized in Table 2. Model details are given in supplementary materials (Table S1), which show that overall, cross-validated accuracy (or classification success) in the model was 75%.

A χ^2^ test (see Table S2) showed that, for those with no *garden or allotment*, 27 out of 28 (*i.e.*, 96% of respondents) had higher stress, whereas, for those with *a garden or allotment*, a lower ratio (51 out of 73% or 70% of respondents) had higher stress.

#### 3.1.3. Predictors of Perceived Stress in Communities 2, 3 and 4

The CCR logistic regression model was run for the combined sample of Communities 2, 3 and 4 (*n* = 305) as for Community 1, using PSS as the dependent variable and independent variables as listed above. The initial regression resulted in four predictors in the model: *place belonging*, *social isolation*, *employment status*, and *car access*, with the two social wellbeing variables occurring in over 99% of “out of sample” regression runs (see Table S3). The absence of environmental variables in a regression run including two social wellbeing variables is perhaps not surprising as the latter are increasingly acknowledged predictors of stress-related ill-health [25,29,30]. In addition, a partial correlation between stress and the green space variables showed some of these were significant when controlling for *place belonging* and *social isolation*. For these reasons, *place belonging* and *social isolation* were removed from the list and the CCR regression run again to explore what other predictors might have been masked by them.

The regression resulted in a model with 9 predictors, as shown in summary in Table 2, with details in Table S4. Next to *car access* and *employment* status, *percentage green space area* contributed most to the model. *Age*, presence of *children in the household*, having *a garden or allotment*, *sex* and *Carstairs score* also contributed to the model. Overall accuracy in the model (or classification success) was 61%. Against expectation, a higher Carstairs score (indicating higher area-level deprivation) was associated with lower stress in this model. However, this variable only contributed 4% to the model and the result may reflect wide individual-level variations in hardship or deprivation masked by the area-level score.

For the green space variables that appeared as significant predictors in this model, a χ^2^ test was run (see Tables S5 and S6). The *percentage green space area* in the neighbourhood contributes 20% to the CCR model and is inversely associated with stress levels (significant at *p* < 0.005): as % green space increases, stress levels decrease. As Table S5 shows, only when the green space area is above 60% is it associated with a greater likelihood of being in the lower, rather than higher, stress group. Having *a garden or allotment* contributes 5% to the model, with 75% of those with access to a garden or allotment having low stress, compared with 52% of those without such access (*p* < 0.01). The link with frequency of winter green space visits also contributed 1% to the model but only approached significance (χ^2^ *p* = 0.056). 

### 3.2. Predicting General Health

#### 3.2.1. Segments in the Sample

**Figure 8:**
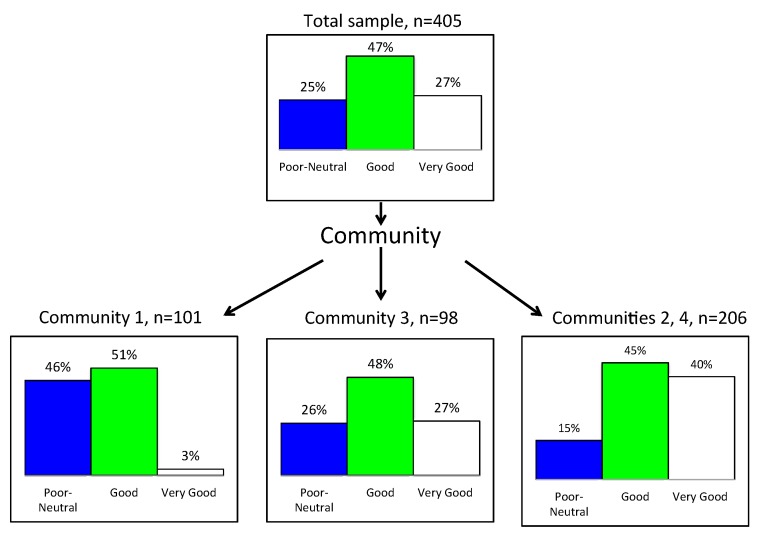
CHAID segmentation of sample by general health.

CHAID was applied to the general, self-report health measure as a three-category variable, distinguishing three groups: those with very poor, poor or “neither poor nor good” health (category 1) from those with good (category 2) or very good health (category 3) (see Figure 8). The top box shows that, overall, 25% of the sample did not have good health while 27% had very good health. Community 1 (*n* = 101) was the least healthy, with 46% of participants not in good health, while Community 3 (*n* = 98) had only 26% not in good health. The Communities reporting best health were 2 and 4 (*n* = 206), with only 15% not in good health and 40% in very good health. The CHAID χ^2^ = 56.98, df = 2, *p* < 0.0001.

#### 3.2.2. Predictors of General Health in Community 1

The CCR logistic regression model for Community 1 used general health as the dependent variable and independent variables as identified earlier. An optimum model was produced with 10 predictors for health. The model is summarised in Table 3 and details are given in supplementary materials (Table S7). Three variables: *physical activity* levels, *social isolation* and *place belonging* contribute most to the model, with a *view of green space or hills from the home* the only green space variable included, contributing 3% to the model. Overall accuracy in the model was 82%.

A χ^2^ test showed that a *view of green space or hills from the home* is associated with better health, with 66.7% of those with such a view having good or very good health, compared with 43.4% of those without a view (see Table S8). This is significant at *p* < 0.03.

#### 3.2.3. Predictors of General Health in Community 3

A single predictor model based on frequency of *winter green space visits* was produced for Community 3. The model is summarised in Table 3 and details are given in supplementary materials (Table S9). Overall accuracy in the model was 73%.

A χ^2^ test (see Table S10) showed that more frequent *winter green space visits* are associated with better health (*p* < 0.02). Only 10% of those who visit green space in winter once a year at most had good health, compared with 35% of those visiting at least once a month in winter.

#### 3.2.4. Predictors of General Health in Communities 2 and 4

The model produced had three predictors: *physical activity*, *age* and *relationship status*. The model is summarised in Table 3 and details are given in supplementary materials (Table S11). Overall accuracy in the model was 70%.

There were no green space predictors in the model. More physical activity was associated with better health, as was lower age. Being single was associated with best health, and being separated or divorced with worst health.

### 3.3. Potential Mediation between Green Space Variables and Perceived Stress

Since social cohesion can mediate the link between green space and health [36], and we observed a significant association between variables for social wellbeing and for green space and perceived stress (in Communities 2, 3 and 4), we decided to explore further the mediating role of social wellbeing in our study. Following a recommendation by Kenny [60], a restricted version of the original Baron and Kenny [61] four-step analysis method was used based on partial correlations.

#### 3.3.1. Percentage Green Space Area and Stress

The intercorrelations between *% green space area*, *place belonging* and *perceived stress* were all significant (see Table S12). Furthermore, the correlation between *place belonging* and *stress* remained significant after controlling for *% green space area* (r = −0.185, *p* < 0.01), indicating that there is a mediating role for *place belonging* between *% green space area* and stress (see Table S13).

The intercorrelations between *% green space area*, *social isolation* and *perceived stress* failed the mediation test, since *% green space area* is not significantly related to *social isolation* (see Table S12).

#### 3.3.2. Having a Garden or Allotment and Stress

The intercorrelations between *having a garden or allotment*, *place belonging* and *perceived stress* were all significant (see Table S12). Furthermore, that between *place belonging* and *perceived stress*, remained significant having controlled for *having a garden or allotment* (r = −0.22, *p* < 0.01), indicating once again a mediating role for *place belonging* (see Table S14).

Intercorrelations between *having a garden or allotment*, *social isolation* and *perceived stress* were also all significant (see Table S12). In addition, the correlation between *social isolation* and *perceived stress* remained significant after controlling for *having a garden or allotment* (r = −0.285, *p* < 0.01), indicating that there is a mediating role for social isolation (see Table S14).

#### 3.3.3. Having a View of Green Space or Hills from the Home and Stress

Since *having a view of green space or hills from the home* was not significantly correlated with *perceived stress*, there can be no mediation role in relation to this variable.

## 4. Discussion

Based on the literature suggesting links between access to local green space and health, particularly levels of stress, in economically deprived communities, we explored potential relationships in our study communities’ data. The sample came from four different urban areas in two of Scotland’s central belt cities−a study across and within cities, which is innovative in design. Although all were in areas of high deprivation, based on Carstairs Index for UK census wards, each site had varying levels of green space and access to private gardens or allotments. We found that there were significant differences by community in levels of self-reported stress and general health, and that measures of green space access were significantly associated with these outcome measures in the majority of cases. Although perceived stress and general health are correlated (*p* < 0.01) in our sample, the predictors for each outcome, including green space variables, were by and large different in each case.

We consider each of the research questions in turn and then relate our findings to the broader hypotheses on which they were based, as well as the limitations of our study.

### 4.1. Characteristics of Access to Green Space Associated with Differences in Perceived Stress Levels

We tested whether characteristics of access to and quantity of local green space are associated with differences in levels of perceived stress in deprived urban communities. We found that having a private garden or allotment and the total green area of space in the neighbourhood (including private gardens) are both predictors of variation in stress levels across all four communities. For Community 1, the community with the highest stress levels (significantly so, with a mean over 5 points higher on the 40-point PSS scale than the other three communities sampled), access to a garden or allotment was the single best predictor of stress levels. For the other three communities, the percentage of neighbourhood (datazone) green space was a significant predictor of stress: an increase in green space was associated with a decrease in stress levels. However, it was only at levels of 60% green space or above that the majority of participants were found to have stress levels below the median.

For most of our participants, having high levels of green space in the neighbourhood, including private gardens as well as public parks, roadside trees and grass or other vegetated areas, appears to be a part of the living environment associated with stress mitigation. The community (Community 1) with the highest levels of self-reported stress did not have the greatest levels of area-level socio-economic deprivation according to the Carstairs Index, but had the lowest levels of educational achievement and highest levels of people not in full-time employment. This is reflected in its 2009 SIMD score [47]. SIMD ranks the 6505 datazones in Scotland, from the most deprived (1) to least deprived (6505), with the most deprived 15% ranked between 1 and 976 [62]. Community 1 had a median of 482, compared to scores of 577, 990 and 543 for Communities 2, 3 and 4, respectively. SIMD was not included in the analysis as it combines measures of health, environment, social and economic context which were being analysed separately. However, the findings from our study support the particularly deprived SIMD measure for Community 1. The significance for stress levels of having access to a private garden or allotment, rather than overall green space, suggests that a green space that offers the potential for personal or private use is important in such a community. 

### 4.2. Physical Activity or Social Wellbeing Variables Associated with Differences in Perceived Stress Levels

We tested whether physical activity or social wellbeing variables were associated with differences in stress levels and whether this association was stronger than for green space variables. Self-reported physical activity levels did not appear in any models predicting stress. However, the social wellbeing variables of social isolation and place belonging were strong predictors of stress in three out of four deprived communities sampled, and it was only after removing these variables that a significant relationship between percentage green space and stress was found. For the third social wellbeing variable, *neighbourhood trust*, mean scores before analysis were similar across communities and no significant relationship between neighbourhood trust and stress was found.

Based on these findings and the associated mediation tests, it appears that green space does not contribute to stress mitigation via enhanced levels of physical activity. Results suggest that its contribution to stress mitigation is partly mediated by its contribution to enhancing *place belonging*, in the case of green space area (*i.e.*, quantity), and to reducing *social isolation*, in the case of access to a garden or allotment. This is supported by ethnographic studies in two of the communities studied [18], showing that green space can offer good opportunities for social contact and for engaging with the local environment, cultural and natural, which enhance a sense of belonging and reduce social isolation. 

In Community 1, the only community where social wellbeing variables were not the strongest predictor of stress, the higher level of multiple deprivation indicated by its SIMD score may reflect poorer social capital in the community as a whole, beyond any socio-economic deprivation indicated by the Carstairs Index. However, given the mediation role identified, it may be that social contact associated with allotment use is an underlying contributor to stress mitigation, as Wood *et al.* [63] suggest in their study demonstrating the mental wellbeing and general health benefits of allotment gardening.

### 4.3. Relationships between Access to Local Green Space and General Health

We tested whether relationships between access to local green space and levels of perceived stress were also found for general health as an outcome. While green space variables appeared as significant in relation to self-reported general health in two of the four communities studied, these were different measures of green space access than those significant for stress.

For one community (Community 3), frequency of green space visits in the winter months of September to March was the best and single predictor of general health. Those who visited at least once a month in winter reported significantly better health than those who did not. Although this community was similar to the others sampled in terms of full-time employment levels, it had the lowest Carstairs index of deprivation and the highest levels of education. We suggest that winter levels of access to green space reflect behaviour by those who access green space all year round, as opposed to those who only visit green space, if at all, in summer months. This association may therefore reflect regular, and comparatively frequent, activity in green space which is beneficial for general health. It may also reflect evidence that exposure to daylight in winter months in northern high latitudes is comparatively more important for general health than in summer months [64].

Green space measures were less important for the other three communities in predicting general health. For two Communities (2 and 4), self-reported physical activity levels were the most important predictor of health, accompanied in the model by age and relationship status. The importance of winter visits, probably reflecting regular year-round visits, to green space, as predicting health in Community 3, may be reflected in Communities 2 and 4 by the variable of physical activity levels. It is possible that physical activity is associated with regular visits to green space in all three communities but predicted less strongly by green space visits in Communities 2 and 4 than in Community 3.

For the community with the worst self-reported health (Community 1), a view of green space or hills from the home was a significant variable in a model predominated by physical activity, as well as measures of social isolation and place belonging. This was also the community with highest perceived stress levels, and both our data and SIMD levels suggest this community may be unusual compared to the others under study. Nonetheless, general health is influenced by psychological as well as physiological factors and our findings would support previous findings that a natural view or distant prospect offers psychological relief that may contribute to health [65], or mitigate ill-health, in deprived communities.

### 4.4. Different Sub-Groups in the Sample

We tested whether there were sub-groups which differ in their pattern of perceived stress and general health in our sample. Our CHAID analysis showed that the primary sub-groups in our data were clusters based on community level differences.

Community 1 stood out as having both worse stress levels and worse general health, despite comparatively high green space area (mean 61.02%), the second highest level for the communities under study. As indicated in Section 2.2, and in data such as employment and educations levels in Table 1, this community has remained an area of very high deprivation despite regeneration efforts over several decades and it may be that the effect of this has led to a lasting stigma of the kind Pearce has identified [66], which continues to affect both health and social outcomes. For this community, access to green space was important both in predicting lower stress (via access to gardens or allotments) and general health (via views to green space or hills from the home). The explanation may lie in the spatial distribution of green space within the community, with most lying in a large unit to the south of the community. Thus, the adjacency of green space to the homes of participants, compared to the quantity present, may be a significant factor in relation to stress and general health outcomes.

For the other communities in our sample (Communities 2, 3 and 4), the level of physical activity is more important for general health in Communities 2 and 4 than any relationship between green space visits and health, unlike in Community 3.

Despite evidence in the literature and previous studies in similar communities that health outcomes, including perceived stress, vary by sex [8], this variable was only found in one of our models, contributing at a low level to predictions of perceived stress in Communities 2, 3 and 4. Given that women in our sample were more likely to report higher stress, further research might usefully explore whether there are sub-groups within such populations for whom green space area and/or social wellbeing variables are differentially associated with stress levels.

### 4.5. Links to Underlying Mechanisms or Pathways

Authors such as Jennings *et al.* [12] have called for more research to investigate the health impacts of the natural environment and, specifically, the cultural role of ecosystem services. Our findings add to understandings of the potential mechanisms or pathways that link quantity and types of access to green space and perceived stress or health levels in deprived urban populations. They suggest the following.

1. The restorative qualities offered by views of green and natural places may contribute to general health (as exemplified in Community 1 by the role of views to green space and hills) via physiological responses that have been demonstrated in a number of studies [4,6,21] The quantity of green space in the neighbourhood, perhaps including views of green space, appears to contribute to place belonging (as exemplified in Communities 2, 3 and 4) and may thereby also contribute to general health, although this link is more tenuous. The fact that percentage of green space was more strongly associated with perceived stress levels than the frequency of visits to green space suggests that it may be green space experienced in moving about the residential neighbourhood while focused on goals other than visiting green space, that is the important factor here. Such findings support earlier studies showing links between chronic stress and percentage green space or natural environment in the residential area [7,8,67].
2. The support that parks, open space and allotments offer for neighbourhood contact and maintenance of community connections, which reduce social isolation and enhance place belonging, may explain the association between the percentage of green space in the neighbourhood and lower perceived stress levels. This confirms earlier findings on links between social cohesion or belonging and residential green space [35,36] and supports evidence from neuroscience that sense of place and place identity, in which the social and natural environment have particular roles, are important dimensions for human health [68]. Future research could usefully distinguish between the value of private gardens *versus* shared gardens and allotments, where greater social interaction with neighbours is likely. The mediating role of social wellbeing variables may explain why, in our study, views of green space or hills from the home were not associated with stress, while percentage of residential green space and gardens or allotments were.
3. The support that parks and open space offer for year-round physical activity and outdoor recreational access, especially in winter months, may explain the relationship between access to green space and general health (as exemplified in Communities 2, 3 and 4). Several studies have shown a positive association between frequency of visits to, and/or time spent in, nearby green space and levels of physical activity [69,70], which in turn predict health [23], although the international evidence for associations between green space and levels of physical activity, remains equivocal, suggesting that the relationship may vary considerably between countries and population sub-groups [37].
4. Of the individual characteristics, the variables associated both with perceived stress levels (in Communities 2, 3 and 4) and general health (in Community 1) were: employment, age, and whether or not there are children under 16 in the household. Unsurprisingly, being in full-time employment was associated with lower perceived stress and better health. However, older age was associated with lower stress but poorer general health. Other studies have shown that older age is associated with an increase in poor health and multi-morbidity, especially among deprived populations [71] but also that the relationship between the amount of green space and health is stronger among older people [72], with a significant association demonstrated between a green environment and levels of physical activity for those aged over 60 years [73]. Future research might usefully explore such interactions further in deprived urban communities. In our study, having children in the household was associated with higher stress but better general health; this latter finding may reflect participants’ younger age where there are children in the household. While caring for children may add to stress in adults, the importance for both parents and children of green space near the home is highlighted in recent research which identifies links between positive birth outcomes and access to green space [74,75], as well as between green space and children’s wellbeing [70,76,77]. Again, future research might usefully explore these interactions in deprived communities such as those of our study.

### 4.6. Limitations

There are a number of limitations to our study. Most importantly, a cross-sectional study is only able to show associations and the direction of causality cannot be determined.

Similar to other research [78], our analyses indicate that the data were strongly clustered around community location although they were intended to be comparable across different communities. This observation implies that community level characteristics are important in understanding the link between green space and our outcome measures. As our focus was on green space, we did not include measures of housing type, age or density in our analysis. These might have associations with health outcomes and suggest potential future avenues that might also be explored. While our data suggest some findings likely to be generalizable to other deprived urban communities in Scotland and possibly other locations in the UK and Europe, they also clearly indicate that local socio-economic and cultural characteristics have a strong influence on perceived stress and general health in the community.

Our measures of green space quantity were based on the most recent and detailed, systematic measures of various types of green and open space area available at the time of study. These data were derived from existing, street level national mapping (Ordnance Survey MasterMap) and detailed mapping of green spaces as part of an audit of the City of Edinburgh. They are now augmented by a national green space map for Scotland [52]. Future such mapping is proposed for England and Wales, but is not readily available for other parts of Europe. Nonetheless, the measures of green space used and what is included or excluded may influence the associations with stress or health in ways that have not yet been examined. We know that ward level census area data on percentage of green space cover yields different figures from those considered at geographic units of datazones [79], and derived units (e.g., 300 m buffers around homes). This reflects the different sources of data (e.g., satellite classification, or detailed scale mapping), the inclusion or exclusion of private gardens, and the different geographies used to summarise spatial data, referred to as the Modifiable Unit Area Problem [80]. Our analysis used what were deemed the most appropriate and accurate measures for our study at the time. However, it may be that further refinement of what is included or excluded in the green space measures, such as seasonally relevant data, yields further understanding.

Measures of the quality of green space were not included in the analysis but might have added predictive power to any associations found. Similarly, analysis of associations between total green space area excluding gardens and allotments was not undertaken but, along with questions on gardening habits of participants, might have yielded more insight into the role of gardening, in particular, in relation to stress. Mapping of the locations of local green space, including gardens and allotments, reported to be used by respondents would have added further understanding of the relevant “neighbourhood” within which such green space is found and the distances people were prepared to travel to access it. This would also indicate to what extent such locations fall within residential datazones. These are fruitful avenues to explore in future research.

## 5. Conclusions

Our study elucidates the relationship between access to green space, perceived stress and general health in economically deprived urban populations. We found that the quantity and nature of access to green space, including specifically access to gardens and allotments and the percentage of all types of green space in the neighbourhood, were significant predictors of stress. For most of our communities sampled, as green space in the neighbourhood increased, levels of perceived stress decreased. The frequency of visits to green space, particularly in winter months, and views of green space from the home were significant predictors of general health, although physical activity was found to be the strongest predictor of general health in half of our sampled communities.

The findings underline the importance of social wellbeing as predictors of stress and, to a lesser extent, general health, offering support for findings by Maas *et al.* [35] and further insight into those of de Vries *et al.* [36]. The variables of social isolation and place belonging were strong predictors of perceived stress in three out of four deprived communities sampled, and of poor general health in the fourth (least healthy) community. There are indications of mediation by social wellbeing in the relationship between local green space and stress levels.

The findings suggest that the contribution of green space to health and wellbeing in deprived communities lies partly in an enhanced sense of place belonging and a reduced sense of social isolation, as well as in offering opportunities to manage or mitigate stress and maintain year-round healthy activity, as indicated by winter visits to green space. In such deprived communities, having access to green space in the neighbourhood may buffer some of the effects of stressors such as unemployment. Factors such as natural space, identity and belonging, and social interaction are all now being taken into account in recently developed public policy towards built and natural environments, such as the Scottish Government Place Standard [9]. The study results have implications for green space standards in urban planning and housing design. For example, in our least healthy community, experiencing greater levels of stress, having access to a garden or allotment was a significant and efficient predictor of perceived stress. In our study, the quantity of all types of green space, public and private, and the ways in which it can be used, appear to make a difference to community-level stress. Encouraging visits to local green space throughout the year also appears to be important for general health.

## Figures and Tables

**Table 1:** **C**haracteristics of the sample (*N* = 406).

Characteristics	Total Sample (*N* = 406)		Community 1 (*N* = 101)		Community 2 (*N* = 100)		Community 3 (*N* = 99)		Community 4 (*N* = 106)	
	%	Mean (SD)	%	Mean (SD)	%	Mean (SD)	%	Mean (SD)	%	Mean (SD)
**Age:**		44 (17.1)		44 (17.0)		42 (16.3)		45 (18.6)		45 (16.5)
16–34	34.6		33.7		37.0		33.7		34.0	
35–54	36.3		34.7		41.0		32.7		36.8	
55–64	11.6		13.9		8.0		14.3		10.4	
65+	17.5		17.8		14.0		19.4		18.9	
**Sex** (% male)	45.3		47.5		44.0		42.4		47.2	
**Education level** (%tertiary+)	14.5		4.0		10.0		28.3		16.2	
**Carstairs Index**		6.5 (2.4)		6.9 (1.0)		8.07 (2.5)		3.7 (0.0)		5.6 (2.6)
**Relationship Status:**										
Single	36.1		25.0		41.0		44.4		34.3	
Married	26.2		27.0		21.0		30.3		26.7	
Partnered/cohabiting	17.5		22.0		20.0		7.1		21.0	
Divorced/separated/widowed	20.2		26.0		18.0		18.2		18.1	
**Employment status:** (% working full-time)	24.6		16.8		20.0		21.2		39.6	
**Children in the household** (%yes)	39.5		46.9		38.9		44.6		28.3	
**Private car access** (% yes)	39.1		23.9		27.3		55.7		48.5	
**Health/Wellbeing**										
**Stress** (PSS score)		15.4 (6.0)		20.0 (1.9)		14.3 (6.4)		13.2 (6.8)		14.0 (5.2)
**General health** (score)		3.9 (1.0)		3.49 (0.7)		4.01 (1.1)		3.85 (1.0)		4.25 (0.8)
**Physical activity** (days/month)		10.3 (10.1)		3.0 (5.8)		12.7 (10.3)		10.0 (9.8)		15.5 (9.4)
**Social wellbeing**										
Place belonging (score)		3.9 (0.9)		3.5 (0.8)		4.1 (1.0)		4.1 (0.9)		4.0 (0.5)
Social isolation (score)		2.5 (0.6)		2.3 (0.6)		2.7 (0.6)		2.5 (0.8)		2.6 (0.5)
Neighbourhood trust (score)		2.9 (1.0)		3.0 (0.7)		2.8 (1.2)		3.0 (1.1)		2.8 (0.8)
**Green space measures**										
% green space area (objective measure)		56.8 (12.3)		61.0 (7.8)		53.5 (6.7)		65.8 (6.7)		49.5 (15.2)
% participants who have a garden or allotment	49.1		72.3		30.0		64.6		30.5	
% participants with view (green space or hill)	30.6		47.5		10.0		42.4		22.9	
% participants visiting green space (at least once a week or more) in winter	57.9		53.0		66.3		68.0		44.4	

Key: **Stress PSS scores:** higher score = greater stress; **General health scores:** higher score = better health; **Place belonging**: higher score = greater place belonging; **Social Isolation:** higher score = less social isolation; **Neighbourhood Trust**: higher score = greater trust.

**Table 2:** Summary table showing predictors of perceived stress (PSS) by Community, based on CCR Logistic regression models and chi-squared tests of significance.

Predictors	Community 1 (Mean PSS Score 20)	Communities 2, 3 and 4 (Mean PSS Score 13.8)	Direction of Relationship between Variables Lower Stress Is Associated with:
Individual characteristics	-	Employment (1) **^a^**,(6) **^b^**	Bring in full-time employment
	-	Car access (4) **^a^**,(5) **^b^**	Having access to a car **^a^**
	-	Age (3) **^b^**	Older age
	-	Children < 16 in household (4) **^b^**	No children in the household
	-	Sex (9) **^b^**	Being male
Area-level deprivation	-	Carstairs Index score (7) **^b^**	Higher area-level deprivation
Social wellbeing	-	Social isolation (3) **^a^**	Not often lacking companionship
	-	Place belonging (2) **^a^**	Greater belonging to the neighbourhood/local area
Green space measures	-	Objective measure of % green space area (1) **^b^**	Greater% green space area
	Garden or allotment (1)	Garden or allotment (2) **^b^**	Having a garden or allotment

**Notes:** Rank order of predictors is shown in parentheses; **^a^** Rank order for first CCR run; **^b^** Rank order for second CCR run with *place belonging* and *social isolation* removed as predictor variables.

**Table 3:** Predictors of self-reported general health by Community, based on CCR Logistic regression models and chi-squared tests of significance.

Predictors	Community 1 (Mean Health Score 3.49)	Community 3 (Mean Health Score 3.85)	Communities 2 and 4 (Mean Health Score 4.13)	Direction of Relationship between Variables Better Health is Associated with:
Individual characteristics	Education level (3)			Higher education level
	Age (5)		Age (2)	Younger age
	Children <16 in household (6)			Children in the household
	Relationship status (8)		Relationship status (3)	Being single
	Employment (10)			Being in full-time employment
Area-level deprivation	Carstairs Index score (7)			Lower area-level deprivation
Physical activity levels (days/month)	Physical activity level (1)		Physical activity level (1)	More days of 30 min or more moderate to vigorous physical activity per month
Social wellbeing	Social isolation (2)			Not often lacking companionship
	Place belonging (4)			Greater belonging to the neighbourhood/local area
Green space measures	View of green space or hills (9)			View of green space or hills from the home
		Winter green space visits (1)		Visiting green space in winter more often (at least once/month)

Note: Rank order of predictors is shown in parentheses.

## Supplementary Materials

The following are available online at www.mdpi.com/1660-4601/13/4/440/s1, Table S1: CCR Logistic regression model predicting PSS for Community 1, Table S2: Chi-squared test showing relationship between PSS and having a garden or allotment, Community 1, Table S3: CCR Logistic regression model predicting PSS for Communities 2, 3 and 4, Table S4: CCR Logistic regression model predicting PSS for Communities 2, 3 and 4, Table S5: Chi-squared test showing relationship between PSS and % green space area, Communities 2, 3 and 4 combined, Table S6: Chi-squared test showing relationship between PSS and access to garden/allotment, Communities 2, 3 and 4, Table S7: CCR Logistic regression model predicting general health for Community 1, Table S8: Chi-squared test showing relationship between general health and view of green space/hills from home, Community 1, Table S9: CCR Logistic regression model predicting general health for Community 3, Table S10: Chi-squared test showing relationship between general health and frequency of green space visits in winter, for Community 3, Table S11: CCR Logistic regression model predicting general health for Communities 2 and 4, Table S12: Significant intercorrelations (Spearman’s rho) between PSS, green space measures and social wellbeing variables, Communities 2, 3 and 4, Table S13: Partial correlation of place belonging with stress, controlling for percentage green space area, Communities 2, 3 and 4, Table S14: Partial correlations of place belonging and social isolation with stress, controlling for having a garden or allotment, Communities 2, 3 and 4.
