# Supplementary material for: Mitigating Stress and Supporting Health in Deprived Urban Communities: The Importance of Green Space and the Social Environment
Source: Int J Environ Res Public Health. 2016 Apr 22;13(4):440. doi: 10.3390/ijerph13040440 (PMC4847102; doi:10.3390/ijerph13040440)
Supplement: Supplementary file 1 [file ijerph-13-00440-s001.pdf]

Supplementary Materials: Mitigating Stress and Supporting Health in Deprived Urban Communities: The Importance of Green Space and the Social Environment

Catharine Ward Thompson, Peter Aspinall, Jenny Roe, Lynette Robertson and David Miller

In the following sections Correlated Component Regression (CCR) data is shown for each of the Community groups in relation to either perceived stress or general health.

In each case, the first table or tables give the model accuracy for both training and cross validation, followed by the standardized regression coefficients and their breakdown by components, and the Pratt Co-efficient.

Subsequent tables show the chi-squared tests for accuracy of prediction of any green space variables shown to be significant.

Table S1. CCR Logistic regression model predicting PSS for Community 1.

**Model Fit**	**Cross-Validation**
**Accuracy**	0.75 (standard error = 0.02)
**AUC (Area under curve)**	0.62 (standard error = 0.03)
**Predictor variables**	**Standardised Coefficient**
Have a garden or allotment [a]	2.69
**Predictor Table**	**Out of sample frequency (n = 3000)**
Have a garden or allotment	2967
**Predictor Importance**	**Contribution to Model (Pratt)**
Have a garden or allotment	100% as only one predictor

Note: [a] 1 = yes, 2 = no.

Table S2. Chi-squared test showing relationship between PSS and having a garden or allotment, Community 1.

**Variable**	**Lower Stress (PSS) %**	**Higher Stress (PSS) %**	**Row Freq (*n*)**
**Have a garden or allotment (before)**			
yes	30	70	73
no	4	96	28
**Total**			**101**

$\chi^2 = 10.39$, df = 1, $p < 0.0013$.

Table S3. CCR Logistic regression model predicting PSS for Communities 2, 3 and 4.

**Model Fit**	**Cross-Validation**		
**Accuracy**	0.72 (standard error = 0.01)		
**AUC (Area under curve)**	0.69 (standard error = 0.02)		
**Predictor Variable**	**Standardised Coefficient**	**Out of Sample Frequency (*n* = 2000 Runs)**	**Contribution to Model (Pratt) %**
In full-time employment [a]	1.09	2000	28%
Place belonging	−0.70	2000	23%
Social isolation [b]	−0.75	1990	30%
Car access [a]	0.82	1960	19%

Notes: [a] 1 = yes, 2 = no; [b] 1 = often, 2 = sometimes, 3 = never.

Table S4. CCR Logistic regression model predicting PSS for Communities 2, 3 and 4.

**Model Fit**	**Cross-Validation**		
**Accuracy**	0.62 (s.e. = 0.01)		
**AUC (Area under curve)**	0.63 (s.e. = 0.01)		
**Predictor Variables**	**Standardised Coefficient**	**Out of Sample Frequency (n = 3000 Runs)**	**Contribution to Model (Pratt) %**
% green space area	−0.59	3000	20
Have a garden or allotment [a]	0.42	3000	5
Age	−0.43	3000	7
Children <16 in household [a]	−0.38	3000	6
Car access [a]	−0.37	3000	28
In full-time employment [a]	0.25	2894	24
Carstairs index	−0.20	2449	4
Frequency green space visit in winter	0.19	2333	1
Sex (0 = male, 1 = female)	0.19	2308	4

Notes: *place belonging* and *social isolation* have been removed as predictor variables, [a] 1 = yes, 2 = no.

Table S5. Chi-squared test showing relationship between PSS and % green space area, Communities 2, 3 and 4 combined.

**Variable**	**Lower Stress (PSS) %**	**Higher Stress (PSS) %**	**Row Freq (n)**
**% green space area (before)**			
24.4–43.7	39.53	60.47	43
44.4–54.0	42.86	57.14	49
54.4–59.3	42.31	57.69	52
60.6–67.5	56.10	43.90	41
69.6–76.3	73.91	26.09	46
**Total**			**231**

$\chi^2 = 15.77$, df = 4, $p < 0.005$.

Table S6. Chi-squared test showing relationship between PSS and access to garden/allotment, Communities 2, 3 and 4.

**Variable**	**Lower Stress (PSS) %**	**Higher Stress (PSS) %**	**Row Freq (n)**
**Access to garden/allotment (before)**			
yes	74.14	25.86	58
no	51.82	48.18	110
**Total**			**168**

$\chi^2 = 8.11$, df = 1 $p < 0.01$.

Table S7. CCR Logistic regression model predicting general health for Community 1.

Model Fit	Cross-Validation		
**Accuracy**	0.82 (s.e. = 0.01)		
**AUC (Area under curve)**	0.87 (s.e. = 0.02)		
**Predictor Variables**	**Standardised Coefficient**	**Out of Sample Frequency (n = 3000 Runs)**	**Contribution to Model (Pratt) %**
Physical activity (days/month)	1.78	2000	20
Social isolation [a]	1.65	2000	19
Education level	1.34	2000	12
Place belonging	1.30	2000	14
Age	−1.13	2000	10
Children <16 in household [b]	−1.09	2000	10
Carstairs index	−1.22	1996	4
Relationship status [c]	0.75	1979	5
A view of green space/hills from home	0.65	1954	3
In full-time employment [a]	−0.60	1834	3

Notes: [a] 1 = often, 2 = sometimes, 3 = never; [b] 1 = yes, 2 = no; [c] 1 = single, 2 = married/partnered/cohabiting, 3 = divorced/separated or widowed.

Table S8. Chi-squared test showing relationship between general health and view of green space/hills from home, Community 1.

Variable	Very Poor, Poor or Neutral (neither Poor nor Good) Health %	Good and Very Good Health %	Row Freq (n)
**View of green space or hills from home (before)**			
no	56.60	43.4	53
yes	33.33	66.6	48
**Total**			**101**

$\chi^2 = 5.62$, df = 1, $p < 0.03$.

Table S9. CCR Logistic regression model predicting general health for Community 3.

Model fit	Cross-Validation		
**Accuracy**	0.73 (s.e. = 0.01)		
**AUC (Area under curve)**	0.58 (s.e. = 0.04)		
**Predictor Variable**	**Standardised Coefficient**	**Out of Sample Frequency (n = 1200 Runs)**	**Contribution to Model (Pratt) %**
Frequency of green space visits in winter	1.49	1740	100

Table S10. Chi-squared test showing relationship between general health and frequency of green space visits in winter, for Community 3.

Variable	Very Poor, Poor or Neutral (neither Poor nor Good) Health %	Good & Very Good Health%	Row Freq (n)
**Frequency of green space visits in winter (before)**			
Never or at least once/year	90	10	31
At least once/month, once/week or daily	65	35	67
**Total**			**98**

$\chi^2 = 6.58$, df = 1, $p < 0.02$.

Table S11. CCR Logistic regression model predicting general health for Communities 2 and 4.

**Model Fit**	**Cross-Validation**		
**Accuracy**	0.70 (s.e. = 0.01)		
**AUC (Area under curve)**	0.77 (s.e. = 0.01)		
**Predictor Variable**	**Standardised Coefficient**	**Out of Sample Frequency (*n* = 2000 Runs)**	**Contribution to Model (Pratt) %**
Physical activity level	1.40	2000	44
Age	−1.17	2000	34
Relationship status [a]	−0.92	1470	22

Notes: [a] 1 = single; 2 = married/partnered/cohabiting; 3 = divorced/separated or widowed.

Table S12. Significant intercorrelations (Spearman's rho) between PSS, green space measures and social wellbeing variables, Communities 2, 3 and 4.

**Variable**	**Statistic**	**PSS**	**% Green Space Area**	**Have a Garden or Allotment**	**Place Belonging**	**Social Isolation**
PSS	Corr coeff	1.000	−0.220 **	0.154 **	−0.250 **	−0.300 **
	Sig. (2-tailed)		0.001	0.007	0.000	0.000
	N	305	231	304	301	303
% green space area	Corr coeff	−0.220 **	1.000	−0.434 **	0.213 **	0.003
	Sig. (2-tailed)	0.001		0.000	0.001	0.962
	N	231	231	231	229	230
Have a garden or allotment	Corr coeff	0.154 **	−0.434 **	1.000	−0.338 **	−0.118 *
	Sig. (2-tailed)	0.007	0.000		0.000	0.041
	N	304	231	304	301	302
Place belonging	Corr coeff	−0.250 **	0.213 **	−0.338 **	1.000	0.090
	Sig. (2-tailed)	0.000	0.001	0.000		0.118
	N	301	229	301	301	300
Social isolation	Corr coeff	−0.300 **	0.003	−0.118 *	0.090	1.000
	Sig. (2-tailed)	0.000	0.962	0.041	0.118	
	N	303	230	302	300	303

* correlation is significant at the 0.05 level (2-tailed); ** correlation is significant at the 0.01 level (2-tailed).

Table S13. Partial correlation of place belonging with stress, controlling for percentage green space area, Communities 2, 3 and 4.

**Control Variable**	**Variable**	**Correlations**	**PSS**	**Place Belonging**
% green space area	PSS	Correlation	1.000	−0.185
		Sig. (2-tailed)		0.005
		df	0	226
	Place belonging	Correlation	−0.185	1.000
		Sig. (2-tailed)	0.005	
		df	226	0

Table S14. Partial correlations of place belonging and social isolation with stress, controlling for having a garden or allotment, Communities 2, 3 and 4.

Control Variable	Variables	Correlations	PSS	Place Belonging
Have a garden or allotment	PSS	Correlation	1.000	−0.22
		Sig (2-tailed)		0.001
		df	0	298
	Place belonging	Correlation	−0.22	1.000
		Sig. (2-tailed)	0.001	
		df	298	0
**Control Variable**	**Variables**	**Correlations**	**PSS**	**Social isolation [a]**
Have a garden or allotment	PSS	Correlation	1.000	−0.285
		Sig. (2-tailed)		0.001
		df	0	299
	Social isolation [a]	Correlation	−0.285	1.000
		Sig. (2-tailed)	0.001	
		df	299	0

[a] a higher score means less social isolation.

© 2016 by the authors; licensee MDPI, Basel, Switzerland. This article is an open access article distributed under the terms and conditions of the Creative Commons by Attribution (CC-BY) license (http://creativecommons.org/licenses/by/4.0/).
